# Pearls in Food

**Published:** 1919-09-27

**Authors:** 


### PEARLS IN FOOD.

IN view of a few somewhat unusual statements which have recently occurred in the daily Press with regard to the finding of pearls in tripe, the following extract from the report of Dr. Hope, Medical Officer of Health for Liverpool, may perhaps with advantage be given. In certain consignments of frozen tripe from the United States there were found scattered over the mucous membranes of some of these stomachs small pearl-like bodies, in some cases very numerous and apparently the result of some parasitic infection. After specimens had been forwarded to Professor Johnstone, D.Sc. of Liverpool University, the following report was made on their structure:

"The material which the Medical Officer of Health sent to me was imported tripe which had been cleaned, prepared, and frozen. The specimens examined contained hundreds of small, iridescent pearl-like bodies, about one thirty-second to one-sixteenth of an inch in diameter. Sections were made, and these were seen to be true pearls, or concretions of soft material (elastic connective tissue) with a very regular laminated structure. Because of the distortion of the tissues caused by boiling and freezing it was difficult to make out much detail."

"The Medical Officer of Health, however, was able to get fresh material (cow's stomach) showing similar bodies. There were true soft pearls embedded in the sub-mucous coat. Each was surrounded by a cyst wall, which consisted of flattened epithelial cells, and which appears to be the formative layer. No nucleus could be seen in these pearl-like bodies. I do not doubt, however, that they orginated round very small parasitic organisms, which may have been sarcosporidia, or perhaps eggs of some worm parasite. These intrusive bodies were probably carried in the blood stream and became arrested in the capillaries of the sub-mucosa of the alimentary canal. Irritation set up a fibrosis round them, which resulted in the establishment of a formative capsule depositing concentric lamellæ of connective tissue round the irritant. The latter, whatever it may have been, is quite destroyed and unrecognisable in the specimens sent. No doubt the condition is the result of an infection. I have seen somewhat similar structures in fish, where the nucleus of the pearl-like body was recognisable as the egg or larva of a cestode parasite."

It is an interesting subject as it stands, and it is almost certain that Dr. Johnstone's explanation will prove correct; but whether the parasite itself, possibly present in other parts of the animal's carcase, will prove to be one of those harmful to man, a well known or a new variety, and, if the latter, whence originated, are all problems which await solution. They are of real importance, not the presence of pearls, as such, in an animal's stomach.

